# Evaluation of the efficacy of ipsilateral fibular transfer for reconstruction of large tibial defects in children: a retrospective study

**DOI:** 10.1186/s13018-022-03021-8

**Published:** 2022-03-05

**Authors:** SiYu Xu, YaoXi Liu, GuangHui Zhu, Kun Liu, Jin Tang, JiangYan Wu, An Yan, Fei Jiang, ShaSha Mo, HaiBo Mei

**Affiliations:** 1 Academy of Pediatrics of University of South China, Department of Pediatric Orthopaedics, Hunan Children’s Hospital, Changsha, 410007 China; 2 Department of Orthopaedics, Dalian Children’s Hospital, Dalian, 116012 China

**Keywords:** Ipsilateral fibula transfer, Fibular centralization, Congenital pseudoarthritis of the tibia, Large tibial defects

## Abstract

**Background:**

Reconstruction of large tibial defects is often a major challenge in limb salvage. This study aimed to evaluate initial follow-up results of ipsilateral fibula transfer for the treatment of large tibial defects in children.

**Methods:**

A retrospective study was performed between September 2014 and April 2021. Ten children were identified as having large tibial defects. The children underwent ipsilateral fibula transfer. We then evaluated initial healing, tibial length discrepancy, ankle varus/valgus, fibular position, refracture, infection, and function.

**Results:**

Five boys and five girls, with an average age of 7.2 years, were evaluated. The transferred fibula was united in the patients. The mean follow-up period after fibular transposition was 43 months. The patients achieved primary bone union; the mean time to union was 8.4 months (range, 4–18 months). Complications included refracture (30%), infection (40%), tibia malunion (30%), ankle varus (30%), sensory loss of toes (10%), and ankle valgus (10%). No other major complications were observed. All 10 patients were able to perform activities of daily living and return to their normal activities.

**Conclusion:**

Ipsilateral fibula transfer is a salvage surgery for the treatment of large tibial defects in children with congenital pseudoarthrosis of the tibia, traumatic nonunion of the tibia, and/or tibial defect after chronic osteomyelitis. However, long-term results still need to be followed up.

## Background

Large tibial defects refer to tibial defects with a length greater than 6 cm [1], which often occur in children with trauma, chronic osteomyelitis, tumours, and congenital pseudoarthritis of the tibia, and other similar conditions [2]. The reconstruction of large bone defects is often a major challenge in limb salvage, regardless of the aetiology of bone loss. In addition, children often have difficulty healing after multiple operations and may eventually require amputation. In recent years, owing to the implementation of new surgical methods, the improved appearance and functional requirements of patients have made limb salvage possible.

The most common and widely accepted procedures for the reconstruction of the tibia are allogeneic bone grafting, vascularised autologous fibular grafting, allogeneic bone composite vascularised fibula reconstruction, distraction osteogenesis, bone transport using the Ilizarov frame, Masquelet-induced membrane technique, and genetic engineering [3–7]. The autologous fibula plays an important role [8–10].

Ipsilateral fibular transfer, developed and popularized by Huntington [11] in the 1940s, is an alternative reconstruction method for limb salvage. Although ipsilateral fibular transfer for the reconstruction of the tibia has been well documented in the treatment of post-traumatic and post-infection tibial defects and some congenital deformities [12–15], to date, few studies have documented the use of this technique in the treatment of congenital pseudoarthritis of the tibia.

In this study, we evaluated the initial clinical efficacy of ipsilateral fibula transfer for the treatment of large tibial defects.

## Methods

Between September 2014 and April 2021, all patients with large tibial defects who underwent ipsilateral fibula transfer were retrospectively reviewed (Tables 1 and 3). The inclusion criteria were as follows: paediatric patients (1) with a diagnosis of large tibial defects, (2) who underwent ipsilateral fibular transfer procedures, and (3) had a minimal follow-up of at least six months. The exclusion criteria were as follows: (1) follow-up of less than six months and (2) incomplete medical records. This study was approved by the Ethics Committee of Hunan Children's Hospital (IORG No. HCHLL-2019-37). The guardians of the included patients signed a written informed consent form prior to participation.

**Table 1:** Patient demographics

Clinical feature	Number of patients (*N* = 10)
Age	
< 10 years	8
> 10 years	2
Previous procedures	
Masquelet procedure	2
Ilizarov procedure	8
Plating	2
Intramedullary nails	5
Bone grafting	7
Primary diagnosis	
Congenital pseudoarthrosis of The tibia	7
Traumatic nonunions of the Tibia	2
Chronic tibial osteomyelitis	1

**Table 2:** Overview of the modified RUST [17]

Score per cortex*	Radiographic criteria		
	Callus	Fracture line	
1	Absent	Visible	Eccentric rod location precludes visualization of cortex
2	Present	Visible	
3	Present	Invisible	Faint lucencies present in dysplastic bone, not representative of fracture line

*Individual cortical scores (anterior, posterior, medial, and lateral) were added to provide a RUST value for a set of radiographs, ranging from 4 (definitely not healed) to 12 (definitely healed). If at least two cortices scored 3, then radiographic healing was considered to have been achieved

**Table 3:** Demographic, operative and outcome details of the patients

Case	Sex/Side	Age at surgery (year)	Tibia lesion/defect before surgery (cm)	Stage procedure	Methods	Surgical time (h)	Blood loss (ml)	Follow-up time (months)	Time before union (months)	Tibia length discrepancy (cm)		TTA (°)		Modified Malhotra grade	Complication (refracture [30%], infection [40%], tibia malunion [30%], ankle varus [30%], sensory loss of toes [10%], ankle valgus [10%])
										Before surgery	last follow-up	Before surgery	last follow-up		
1	M/R	3.4	6.4	1	1 + 2 + 3 + 4	4	80	51	12	11.1	4.6	− 12.8	7.1	− 3	Left hip incision infection, valgus ankle
2	F/L	11.3	6	1	1 + 2 + 3 + 4	5	100	27	7	9.2	9	− 12.6	3.9	0	Refracture
3	M/R	5.8	9	1	1 + 2 + 3 + 4 + 5	4	100	7	4	14.9	7.2	11.5	10.8	− 1	Right calf incision, left hip incision infection
4	F/L	9.9	8	1	1 + 2 + 3 + 4	2	220	48	7	19.1	6.5	− 3.2	3.7	**0**	Refracture
5	F/L	16.5	8	1	1 + 4	5	50	9	9	19	7	− 5.9	6.6	0	Sensory loss of toes
6	M/R	1.3	4.6	1	1 + 2 + 4 + 6	4.5	100	75	5	6	1.9	0	4.9	0	Pin-tract infection, posterior arch of proximal tibia
7	M/R	9	6.3	2	1 + 2 + 5	3/4	20/60	78	7	16.4	0.2	3.5	− 3.9	+ 1	Anterior arch of distal tibia, varus ankle
8	M/R	7.6	7.2	2	1 + 2 + 3 + 4	4/2	60/20	31	18	17.9	6.8	15	13.7	− 2	Pin-tract infection, refracture
9	F/R	2.1	14.7	2	1 + 2 + 3 + 4	2.5/3	20/50	32	10	16.2	2.8	3.5	0	+ 1	Varus ankle
10	F/R	5	14.7	1	1 + 2 + 3 + 4	4	110	72	5	18.5	0	10	− 10	+ 1	Posterior arch of proximal tibia, varus ankle
Average		7.2	8.5			4.2	99	43	8.4	14.8	4.6	0.9	3.7		

Methods: (1) Ipsilateral fibula transfer, (2) Wrapped bone graft, (3) Shifted fibula Kirschner wire internal fixation, (4) Ilizarov fixation, (5) Long leg cast, and (6) Intramedullary rod fixation via the ankle. M male, F female, L left, R right

### Operative technique

#### Harvesting autogenic iliac bone

**Fig. 1:**
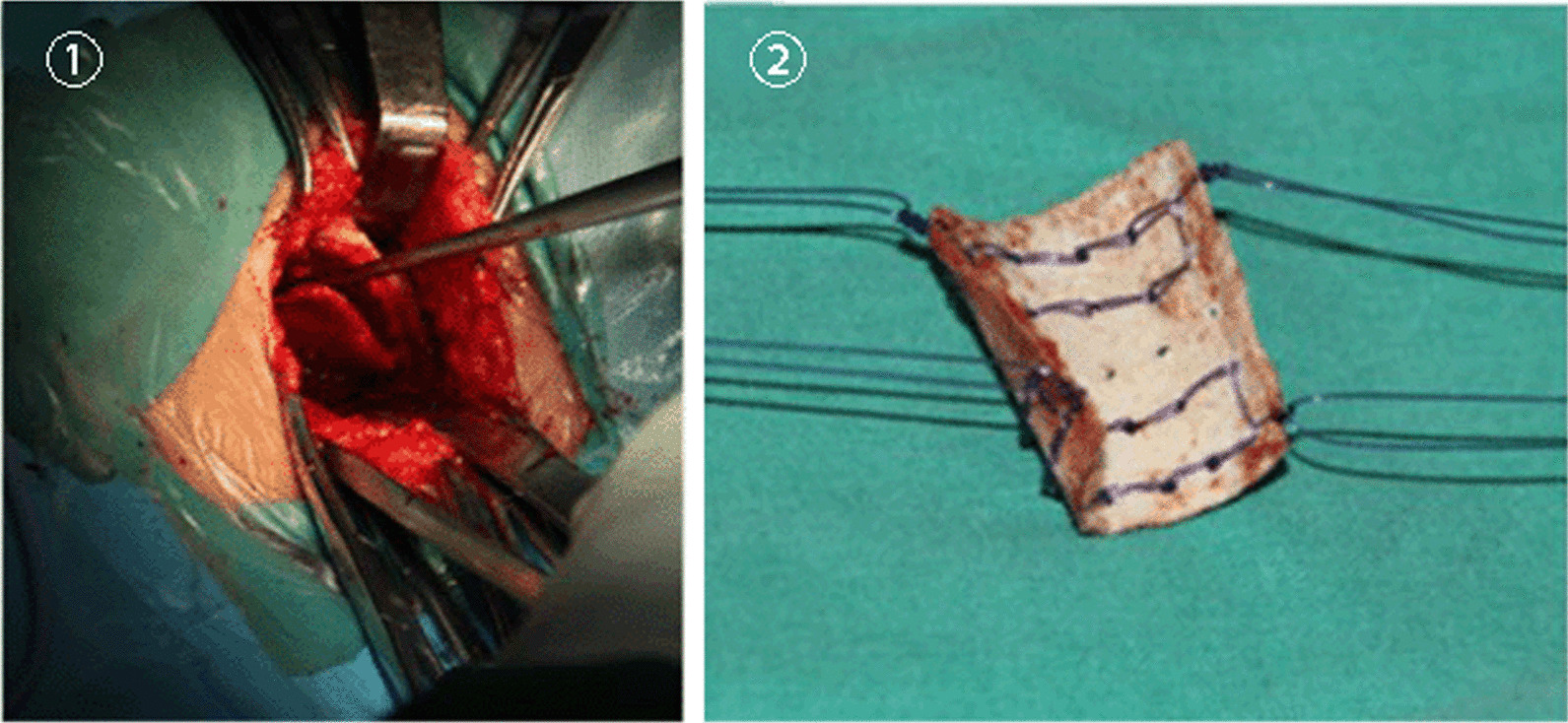
Harvesting and suturing autogenic iliac bone. Exposure of the outer table of the ilium, harvesting a rectangular cortex. Holes were made in the rectangular cortex with a Kirschner wire and with doubled absorbable sutures on each corner. The rectangular cortex was bent to produce a cylindrical shape for the wrapping of the cancellous bone graft [16]

The iliac bone graft was harvested through a straight incision centred over the anterior superior iliac spine. The apophysis of the ilium was split, and the outer table of the anterolateral surface of the ilium was exposed subperiosteally. A rectangular cortex was obtained from the outer table of the ilium while keeping the inner wall intact. Several holes were made in the rectangular cortex with a 1.5-mm Kirschner wire. The bone was sutured with absorbable sutures at each corner and bent into a cylindrical shape (Fig. 1).

### Transposition

#### Fibular transfer at the proximal level

In total, 2–3 cm of the proximal fibula was exposed subperiosteally above the level of the distal part of the proximal tibial fragment. The distal part of the proximal fragment of the tibia was exposed subperiosteally, and a trap-door entry was made into the distal part of the medullary canal. The upper end of the exposed fibula was divided. When intramedullary fixation was anticipated, pre-reaming of the distal fragment of the fibula was performed. The proximal end of the distal fragment of the fibula was then transplanted into a slot made in the proximal segment of the tibia. The fibula was fixed to the tibia over the closed trap-door using a Williams’ rod/ K-wire, the length and diameter of which were preoperatively determined using digital radiography and introduced through the medial side of the tibia into the distal segment of the fibula.

#### Fibular transfer at the distal level

The subperiosteal fibula was cut at the level of the distal tibia defect, and the periosteum of the fibula was peeled off. The distal tibia was linked to the proximal fibula, and the proximal fibula was inserted into the distal medullary cavity of the tibial defect for fixation. A Williams’ rod/K-wire, the length and diameter of which were preoperatively determined using digital radiography, was inserted into the medullary canal of the fibula across the distal tibia, from the proximal to distal direction via the calcaneus and talus and out of the sole through the heel pad. Fluoroscopy was used to ensure that the Williams’ rod/K-wire was located in the centre of the distal tibial physis on anteroposterior and lateral views, and a neutral dorsiflexion-plantar flexion of the foot and neutral varus-valgus alignment of the ankle were maintained. The Williams’ rod/K-wire was then driven retrograde into the proximal tibiofibular fragment, which was anatomically aligned in both the coronal and sagittal planes. This placement was verified using intraoperative imaging.

#### Fixation

Ilizarov fixator was mounted with one full ring above the site of the tibiofibular fragment and one full ring below. Both rings were fixed with two or three 1.5- or 2.0-mm tensioned Kirschner wires through the tibia and were connected together by threaded rods, subsequently applying appropriate pressure at the site of the tibiofibular fragment.

#### Wrapping autogenic iliac bone graft

**Fig. 2:**
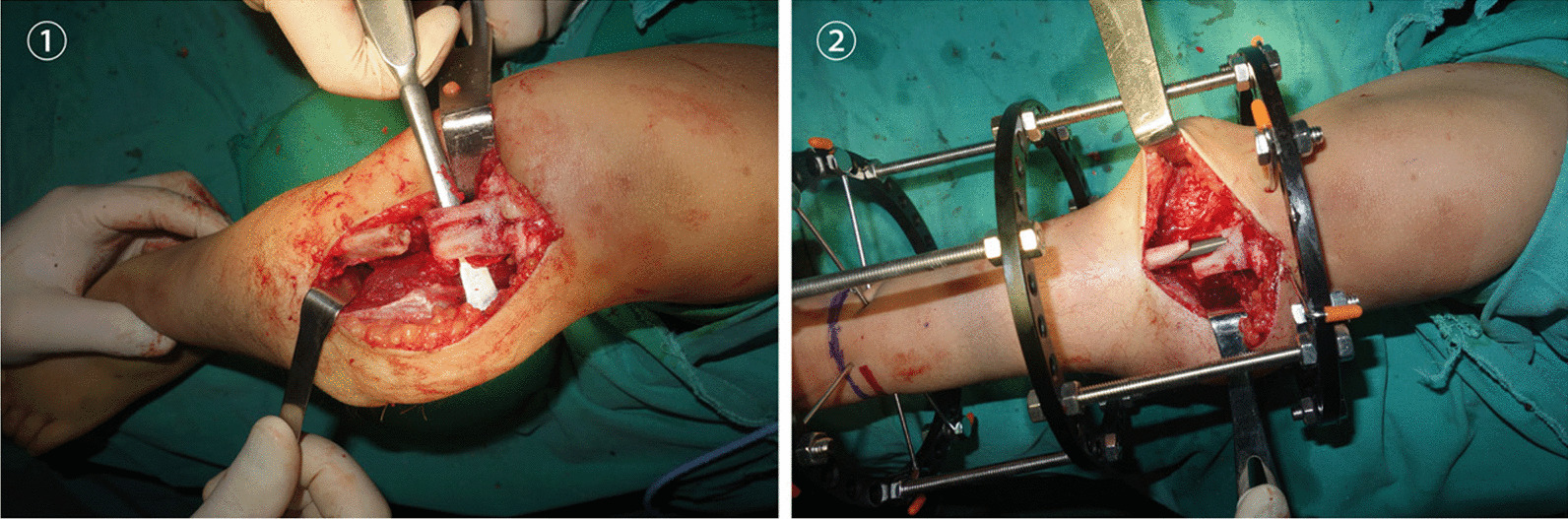
Fibular transfer at the proximal level

The previously harvested cylindrical cortex was wrapped around the site of the tibiofibular fragment after the application of the Ilizarov fixator. The cortex was tied with absorbable sutures, which had previously been connected to the cortex, establishing a sealed environment for the enhancement of osteogenesis (Fig. 2) [16].

#### Postoperative period

Patients were followed up every two months until radiographic union was obtained. The time to union of the proximal and distal ends of the transplantation area, the length of the tibial defect, the modified Malhotra grade, and the tibiotalar tilt angle (TTA) were evaluated by ascertaining whether the bones had healed clinically and radiographically, whether the infection had been eradicated, and whether a refracture had occurred. In addition, the knee and ankle ranges of motion, tibial length, and whether patients were able to perform activities of daily living and work were recorded. At the most recent follow-up, 7–78 months after the success of the operations, the patients were asked if they would have preferred the option of amputation and life-long prosthesis; none said that they would have preferred amputation.

## Evaluation index

**Fig. 3:**
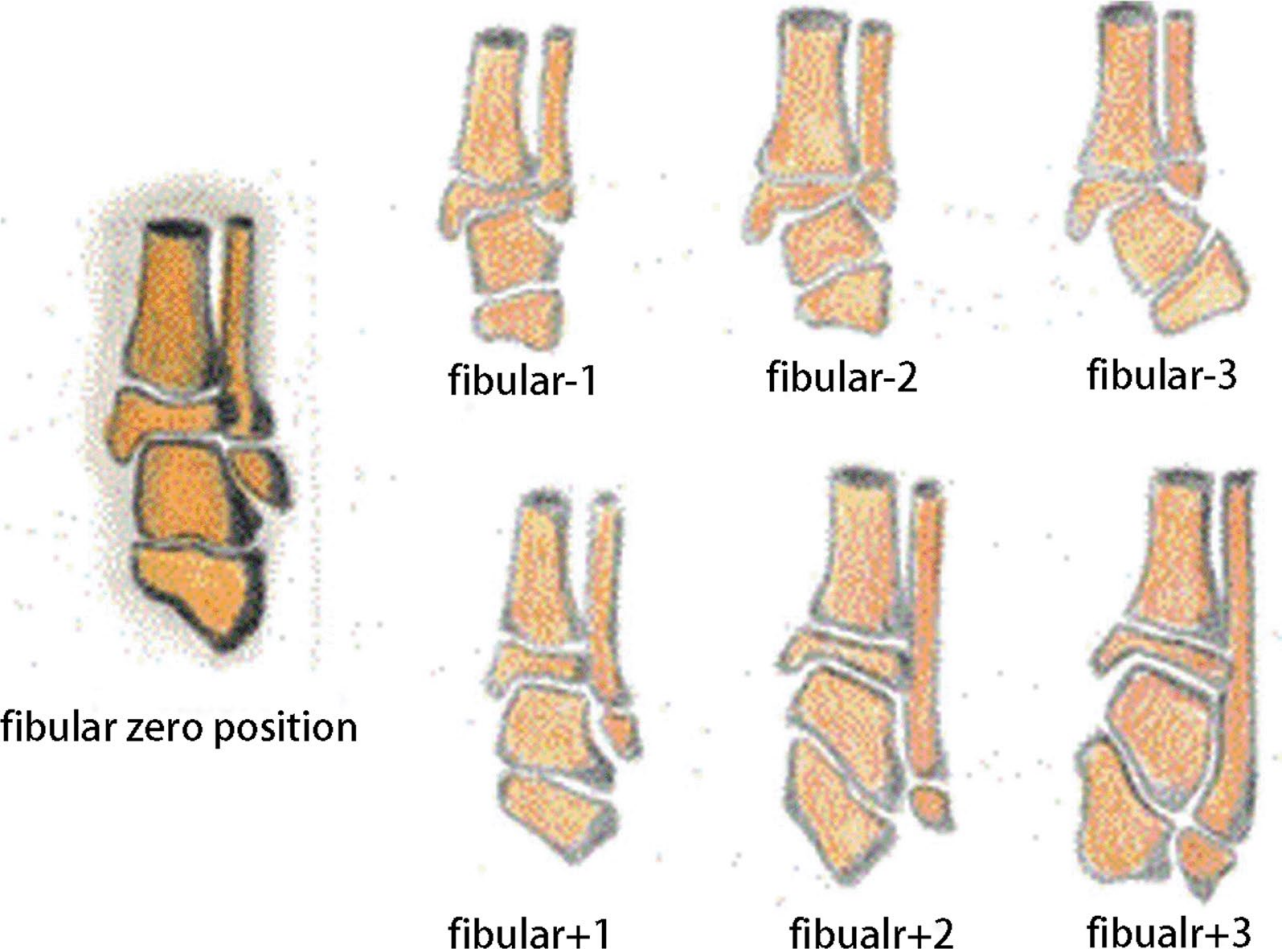
Illustrations showing the Elgohary and Elmoghazy scale, a modification of the Malhotra scale [20]

1. Evaluation method for bone healing at the tibiofibular fragment (refer to the scoring standard for judging bone healing of congenital pseudoarthrosis of the tibia): According to the modified radiographic union scale in tibial (RUST) fractures scoring method, a score greater than 8 indicates bone healing [17] (Table 2).
2. Tibial length: The length of the contralateral tibia is the distance from the midpoint of the proximal tibial physis to the midpoint of the distal tibial physis. The length of the lesion or defect of the ipsilateral limb can be directly measured, and the total length of the remaining healthy tibia is the sum of the distance from the midpoint of the proximal physis to the proximal end of the healthy tibia and the distance from the distal end of the healthy tibia to the midpoint of the distal physis. The tibial defect length is the length of the contralateral tibia minus the length of the healthy tibia on the ipsilateral side.
3. TTA [18]: The change in the ankle joint is determined by measuring the TTA, which is defined as the angle formed by a line perpendicular to the longitudinal axis of the tibia and a second line drawn across the dome of the talus. The normal range in children has been reported to be 0° to 3° valgus.
4. Fibular position: The fibular position was evaluated according to the Elgohary and Elmoghazy scale, a modification of the Malhotra scale (Fig. 3) [19, 20]. Zero position (0) is the normal position, with the distal fibular growth plate and the talar plateau levelled. The fibula minus 1 position (fibula − 1) is where the distal fibular growth plate lies between the top of the talus and the distal tibial growth plate. The fibula minus 2 position (fibula − 2) is where the distal fibular growth plate is levelled with the distal tibial growth plate. The fibula minus 3 position (fibula − 3) is where the distal fibular growth plate lies proximal to the distal tibial growth plate. The fibula plus 1 position (fibula + 1) is where the distal fibular growth plate lies between the talar plateau and the middle of the lateral surface of the talus. The fibula plus 2 position (fibula + 2) is where the distal fibular growth plate lies between the middle of the lateral surface of the talus and above the level of the talocalcaneal joint. The fibula plus 3 position (fibula + 3) is where the distal fibular growth plate lies at or distal to the level of the talocalcaneal joint.

## Results

We enrolled 10 patients with large tibial defects treated via ipsilateral fibula transfer. The mean follow-up period was 43 months (range, 7–78 months). The mean age at the time of operation was 7.2 years (range, 1.3–16.5 years). The patients previously underwent two to four operations. The primary disease included seven cases of congenital pseudoarthrosis of the tibia accompanied by type I neurofibromatosis (NF-1), all of which were Crawford IV. Three of these cases were accompanied by fibular pseudoarthrosis, while the remaining four cases were not. Two patients were diagnosed with traumatic nonunion of the tibia. One patient was diagnosed with chronic tibial osteomyelitis. These three cases were not accompanied by NF-1 or fibular pseudoarthrosis. The average preoperative length of the tibial lesion or defect on the affected side was 8.5 cm (range, 4.6–14.7 cm). The ipsilateral tibia was shorter than the opposite side by 14.8 cm (range, 6–19.1 cm). During the follow-up, primary bone union was achieved in all 10 patients, with an average healing time of 8.4 months (range, 4–18 months).

At the recent follow-up, the postoperative modified Malhotra grades were as follows: fibula + 1 (*n* = 3), fibula − 1 (*n* = 1), fibula − 2 (*n* = 1), fibula − 3 (*n*  = 1), and fibula 0 (*n* = 4). Three patients (30%) with a fibula + 1 position were complicated with a varus deformity of the ankle. One patient (10%) in the fibula − 3 position was complicated with a valgus deformity of the ankle. Ankle deformities may need to be corrected in the long term. The average preoperative TTA was 0.9° (range, − 12.8° to 15°) and 3.7° (range, − 10° to 13.7°) at the last follow-up; the TTA of seven patients (70%) was over 3°. The average tibial length discrepancy at the last follow-up was 4.2 cm (range, 0.2–6.8 cm). The transferred fibula in three patients (30%) were refractured at postoperative 7, 15 and 31 months, respectively. Two cases of refracture healed after secondary surgery, and one patient had been surgically treated for two months at the time of writing. One patient (10%) developed a left iliac wound infection, which was cured by debridement, vacuum sealing drainage, and gluteus maximus muscle flap packing. One patient (10%) developed an infection of the right calf incision, which was cured using antibiotics. Two patients (20%) had pin-tract infections, which were cured using a pin-tract nurse and oral administration of antibiotics. Two patients (20%) developed a posterior arch of the proximal tibia, and one (10%), an anterior arch of the distal tibia, all of them were cured using orthopaedic surgery. One patient (10%) developed loss of sensation on the toes, which may have been caused by cutaneous nerve injury. There were no other complications, such as knee contracture, ankle joint stiffness, or adjacent joint pain.

The range of motion of the knee in one case was limited to 5°–30°. Nine patients achieved nearly full range of motion of the knee joint, and three patients achieved full range of motion of the ankles. The range of motion of the ankles of seven patients was limited to varying degrees, limb shortening ranged from 0 to 9 cm, and the patients had an increase in the use of their footwear. Nine patients were able to perform activities of daily living using a leg brace. One patient wore an external fixator and was prohibited from weight-bearing activities; thus, he had to use a wheelchair. All 10 patients were able to perform activities of daily living and return to their normal activities: nine as students, and one as a homemaker. The walking distances were as follows: < 100 m (*n* = 1), 100–500 m (*n* = 0), and 500 m–1.5 km (*n*  = 9).

Single-stage procedures were performed in seven patients. Here, fibular transfer was performed at the proximal level only because during follow-up, the distal site underwent spontaneous synostosis, and there was remarkable regeneration of the distal half of the tibia. In 3 of the 10 patients, ipsilateral fibular transfer was performed in two stages because of the difficult mobilization of the fibula.

**Fig. 4:**
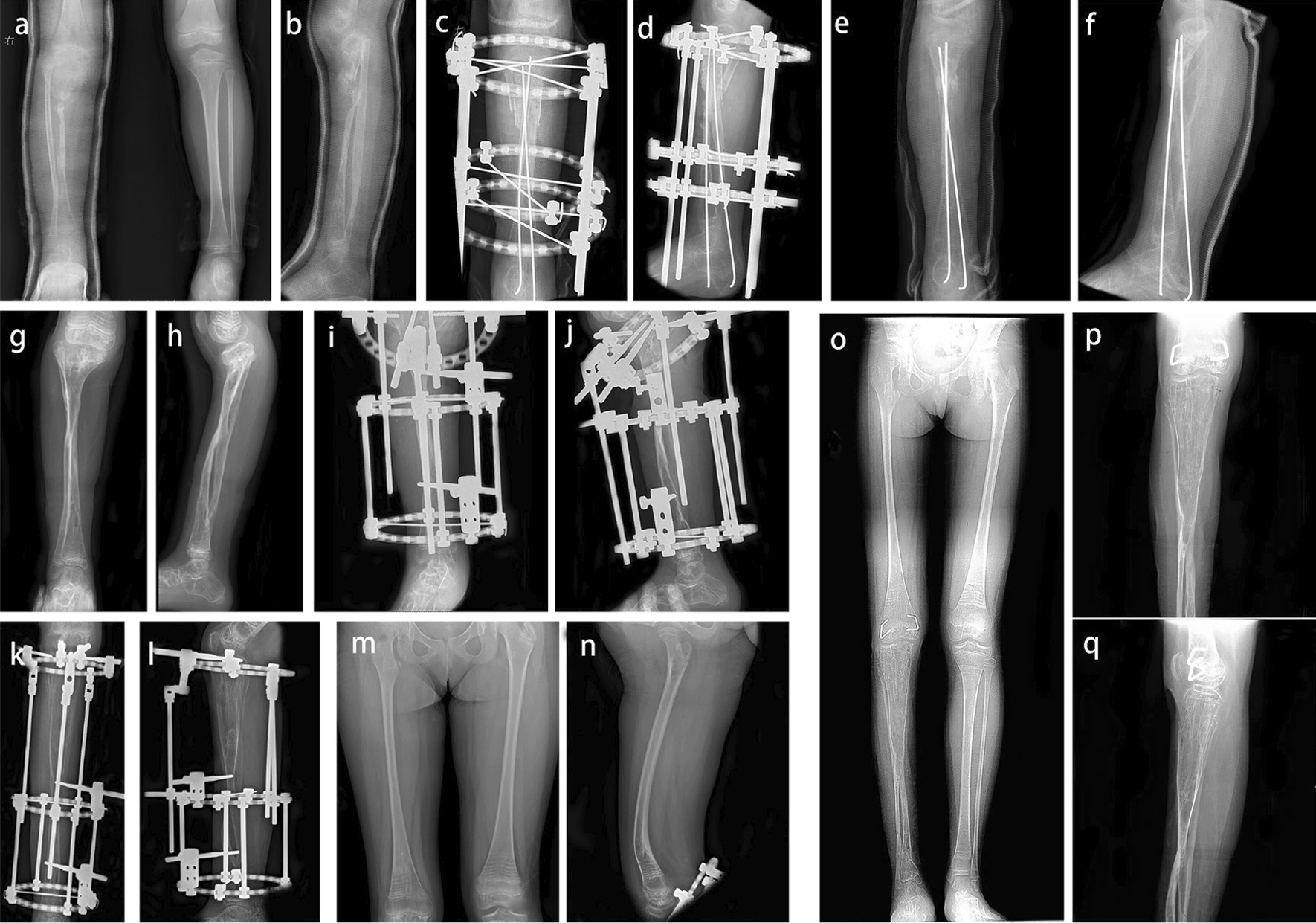
Case 10: Radiographic presentation of a 5-year-old girl with right congenital pseudoarthrosis of the tibia treated via debridement, autogenous bone grafting, and external fixation prior to ipsilateral fibula transfer. **a**, **b** Preoperative anteroposterior and lateral radiographs: The tibial lesion was approximately 14.7 cm, and the remaining normal tibia was shorter than the contralateral side by approximately 18.5 cm. **c**, **d** Postoperative radiograph with ipsilateral fibular transfer. **e**, **f** 5 months after the ipsilateral fibular transfer. **g**, **h** 3.7 years postoperative follow-up, a deformity of the posterior arch of the proximal tibia can also be seen. **i**, **j** Due to the proximal posterior arch of the tibia, she underwent an oblique osteotomy and a gradual lengthening of the fibula via external fixation. **k**, **l** Radiographs were again taken 1.3 year after the proximal epiphysis osteotomy and fixation. **m**, **n** 7 months after the fracture of the right distal femur, the distal femur showed an anterior arch deformity. **o**–**q** 6 years postoperative follow-up, significant hypertrophy of the transplanted fibula can be seen

**Fig.5:**
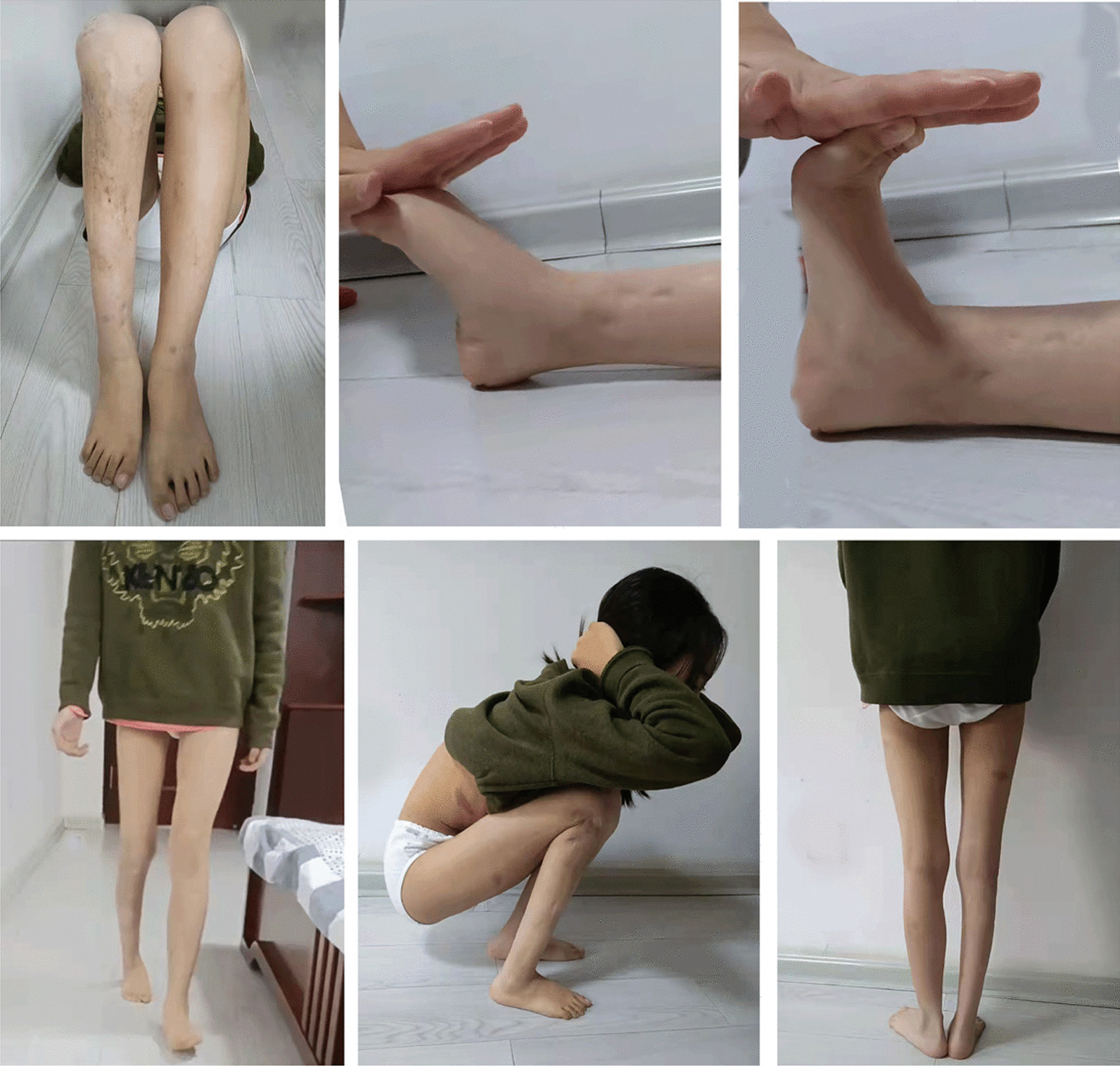
Case 10: Function of the lower limbs

Demographic data of the participants are summarized in Table 3. A typical case is presented in Fig. 4 and the function pictures in Fig. 5.

## Discussion

Treatment of major bone gaps is demanding, and if patients are not willing to undergo prolonged treatment, amputation remains the only option. The severity of soft tissue loss and insensate feet are the most important reasons for the consideration of amputation. However, compared to limb salvage in the long run, the cost of the maintenance of prostheses is considerably higher than the cost of successful reconstruction [21]. Significant segmental defects of the tibia can be reconstructed via bone transport using the Ilizarov frame, Masquelet-induced membrane technique, and bone grafting. Several new techniques have been developed to treat this condition, such as the Papineau technique, three-dimensional printing prosthesis, and tissue engineering [3, 5, 6]. Lengthening beyond 25% of the original segment length is an orthopaedic challenge, and substantial lengthening is fraught with complications, such as joint contractures, refractures, nerve injuries, and prolonged periods of consolidation [22–25]. The Masquelet-induced membrane technique has some serious complications, including bone graft resorption and delayed stress fracture. Additionally, the affected limb will likely eventually develop into a large bone defect. Two cases in this study were previously using the Masquelet technique. The first stage of the operations was successful. Unfortunately, bone graft resorption occurred after the second stage, and a large tibial bone defect redeveloped, indicating that the reconstruction operation had failed.

Albert first presented the use of a fibula to replace the tibia in 1877. He successfully treated a patient with a defect in the distal tibia; since then, the technique has been continuously explored and developed [6, 8, 17, 23, 24]. Ever since Taylor used the contralateral fibula to successfully treat a post-traumatic tibial defect in 1973 [8], vascularised fibula grafting has gradually become a new method to repair long bone defects, and there have been many reports of successful clinical applications. This kind of fibular grafting with a blood supply does not require a ‘creeping replacement’ process, and the healing rate is high. Moreover, the grafted vascularised fibula has a strong ability to survive and adapt to the new biomechanical environment. Through gradual weight-bearing of the lower limbs with the stimulation of stress, the fibula can gradually thicken until it replaces the tibia [9]. However, vascularised fibular transfer requires sophisticated equipment and expertise in the microvascular field, which is difficult to carry out on a large scale in clinical situations. The vascular diameter in children is small, and due to inflammatory stimuli, the affected area is often full of scar tissue. The blood vessel wall is fragile, which increases the difficulty of vascular anastomosis. It is easy for vasospasm, thrombosis, blood circulation disorders, and pain to develop. Ankle instability, peroneal nerve injury, and progressive ankle valgus can be observed in the long term [22].

The transposition of the ipsilateral fibula to tibial defects was first proposed by Hahn in 1884 and was later used successfully by Huntington in 1903 [11]. Huntington and Tuli [14] transferred a fibula in two stages, while Catagni [26] described a single-stage end-to-end apposition of the fibula, which not only guarantees blood supply, but also combines the advantages of fibula transplantation [9]. The difficulty of surgery is less than that of vascularised fibula transplantation.

Therefore, when other surgical methods have failed to treat a large tibial defect with a complete ipsilateral fibula, or when the patient is unwilling to cut the graft bone tissue from the healthy side, the transfer of the ipsilateral fibula may be an option apart from amputation [14, 27]. We transferred the ipsilateral fibula and placed it in an intramedullary location. When the graft is located on the mechanical and anatomical axis of the tibia, the risk of fracture is reduced [28]. The vascularised fibula heals through the bone and does not require ‘creeping replacement’, which can occur in vascularised grafts, and has all the advantages of a vascularised fibula graft without the requirement of microvascular expertise. A shorter operation time may help reduce infection. The fibula has a rich blood supply from the peroneal artery nutrient branch and surrounding muscle attachments, which leads to early union, and good vascularity can reduce the risk of infection. Therefore, the fibula is a good choice for the reconstruction of tibial defects because it has good mechanical properties and the ability to become hypertrophied. The fibula has great potential for remodelling and hypertrophy when subjected to a continuous mechanical load (Wolfe's Law) [28].

The indications for Huntington's procedure are large defects of the tibia (due to trauma, tumour, pseudoarthrosis, and osteomyelitis) accompanied by scars, infections, severe soft tissue injuries, malalignment of the limb, and failure of conventional techniques [21]. The management of large defects of the tibia in the aforementioned scenario is difficult when conventional methods are used, such as bone transport, induced membrane technique, auto/allografting, distraction osteogenesis, and microvascular surgery. The contraindication of Huntington's procedure includes multilevel fibular fractures and loss of peroneal vessels due to trauma.

This technique has a wide range of clinical applications. Shrivastava [29] presented a variant of tibial hemimelia in 2009 by centralizing the fibula and obtained satisfactory results. A previous study reported a one-year healing rate of 75% [13]; they used ipsilateral fibular transposition in four patients with tibial defects ranging from 5 to 20 cm. One valgus deformity were noted, and none of the patients had symptoms related to tibiofibular synostosis. Another study treated 18 children with complex gap nonunion of the tibia with an average bone defect of 6.6 cm (range, 5–17 cm) using the Huntington procedure [10]. The average time to union was 11.5 weeks and 13.8 weeks for proximal and distal tibiofibular synostosis, respectively. Hypertrophy of the fibula was observed in all patients. One patient developed ankle varus, and three had ankle equinus. Nine patients developed flexion deformity of the knee and two patients had foot drop.

We used ipsilateral fibula transfer to treat children with large tibial defects for different reasons. Based on the current short-term follow-up results, 10 cases achieved initial bone union according to the modified RUST score, and the mechanical and anatomical axes were basically normal, which is consistent with a previously reported long-term follow-up report [27]. However, during the follow-up period, several cases presented with some complications. These included refracture (30%), infection (40%), tibia malunion (30%), ankle varus (30%), sensation of the loss of the toes (10%), and ankle valgus (10%).

A previous study pointed out that proximal tibial dysplasia reflects a pathological process of the periosteum, similar to fibrous hamartoma in the pseudarthrosis site, and that the extent of this lesion varies among patients [30]. Proximal tibial dysplasia may result in an unbalanced growth of the proximal tibia. If there is no timely intervention, refracture may occur in the long term. Some complications were corrected after further treatment; this issue also needs to be addressed to during the application of this surgical method. One of the limitations of ipsilateral fibula transfer is that it may not provide acceptable limb alignment in all patients. This was the source of ankle deformity in four of our patients. Ipsilateral fibular transfer avoids exposure of the site that is vulnerable to infection and there is also debulking of the leg that facilitates wound closure and subsequent soft tissue healing. Both these factors are helpful in reducing postoperative infection. Despite this fact, four of our patients developed infection. The cause of sensory loss of the toes was iatrogenic common peroneal nerve traction injury in one of our patients while attempting to centralize the fibula into the medullary canal of the tibia. Therefore, the authors suggest that the fibula should not be overzealous in cases with extensive soft tissue fibrosis.

We anticipate the success of replacing the tibia with the fibula on the affected side and correcting the tibial defect. The fibula can recover through functional exercise to stimulate fibular thickening and tibialization of the fibula combined with the Ilizarov frame for distraction osteogenesis to extend the centred fibula, resulting in a satisfactory shape and function of the lower limbs.

However, fibular grafts on the ipsilateral side are sometimes difficult to obtain because the donor site is in the same injured leg or infected site. The fibula may be complicated by multiple fractures or vascular and nerve damage, and a sufficient length of the fibula may not be obtainable. Although three patients in our study had fibular pseudoarthrosis, the lesions were located at the distal end of the fibula. A sufficient fibular length can still be obtained, and the limitations of the procedure include primary applicability in young patients because the rate of union is faster and hypertrophy is maximized in younger patients. The limitations of the procedure include its inability to effectively address limb length discrepancy and occasional deformity. These complications may require additional surgery to improve the outcomes.

The present study had four limitations. First, the follow-up period was not long enough to document the real incidence of residual deformities with this approach, including proximal tibial valgus, ankle deformity, and adjacent joint dysfunction. It is necessary to follow-up on these younger patients until they reach skeletal maturity and to evaluate the long-term outcome of the ipsilateral fibular transfer technique, such as hypertrophy of the fibula. Second, due to the retrospective nature of the study, some data are not available for comparative research of this series. Third, it lacks function and statistical analysis comparisons with other methods. Last, because of the rarity of the disease, this study was limited by the small sample size. A large number of cases could have resulted in different results especially in patients’ demographics, surgical outcomes, and complications. Further studies with more patients, comparisons with other methods, and long-term follow-up are necessary.

## Conclusion

Ipsilateral fibular transfer may be considered as a simple and cost-effective surgical method for the treatment of large tibial defects. It does not require microvascular expertise and implants, does not lead to donor site complications, can be performed under appropriate conditions, can be used to replace the tibia, and has a shorter recovery period than other techniques. In some special cases, this is a reasonable choice for limb salvage surgery in the treatment of large tibial defects. However, residual deformities, such as refracture, tibial deformity, ankle deformity, nerve injury, and adjacent joint dysfunction, can also develop and should be given more attention. It is necessary to follow-up on these patients until they reach skeletal maturity and evaluate the long-term outcomes of the ipsilateral fibular transfer.

## Data Availability

The datasets used and/or analysed during the current study are available from the corresponding author on reasonable request.

## Abbreviations

TTA: Tibiotalar tilt angle
RUST: Radiographic union scale in tibial
NF-1: Type 1 neurofibromatosis
